# Establishment and Characterization of a Sclerosing Spindle Cell Rhabdomyosarcoma Cell Line with a Complex Genomic Profile

**DOI:** 10.3390/cells9122668

**Published:** 2020-12-11

**Authors:** Sabine Schleicher, Stefan Grote, Elke Malenke, Kenneth Chun-Ho Chan, Martin Schaller, Birgit Fehrenbacher, Rosa Riester, Torsten Kluba, Leonie Frauenfeld, Hans Boesmueller, Gudrun Göhring, Brigitte Schlegelberger, Rupert Handgretinger, Hans-Georg Kopp, Frank Traub, Karen A. Boehme

**Affiliations:** 1 Department of Hematology and Oncology, Eberhard Karls University Tuebingen, Children’s Hospital, 72076 Tuebingen, Germany; stefan.grote@med.uni-tuebingen.de (S.G.); elke.malenke@med.uni-tuebingen.de (E.M.); KC220@outlook.com (K.C.-H.C.); rupert.handgretinger@med.uni-tuebingen.de (R.H.); 2 Department of Dermatology, Eberhard Karls University Tuebingen, 72076 Tuebingen, Germany; martin.schaller@med.uni-tuebingen.de (M.S.); birgit.fehrenbacher@med.uni-tuebingen.de (B.F.); 3 Department of Orthopaedic Surgery, Eberhard Karls University Tuebingen, 72072 Tuebingen, Germany; rosa.riester@med.uni-tuebingen.de (R.R.); karen.boehme@web.de (K.A.B.); 4 Department for Orthopaedic Surgery, Hospital Dresden-Friedrichstadt, 01067 Dresden, Germany; torsten.kluba@khdf.de; 5 Institute of Pathology and Neuropathology, University Hospital Tuebingen, 72076 Tuebingen, Germany; leonie.frauenfeld@med.uni-tuebingen.de (L.F.); hans.boesmueller@med.uni-tuebingen.de (H.B.); 6 Department of Human Genetics, Hannover Medical School, 30625 Hannover, Germany; goehring.gudrun@mh-hannover.de (G.G.); schlegelberger.brigitte@mh-hannover.de (B.S.); 7 Robert Bosch Cancer Center/RBCT Stuttgart, 70376 Stuttgart, Germany; hans-georg.kopp@rbk.de; 8 Department of Orthopedics and Traumatology, University Medical Centre Mainz, Johannes Gutenberg-University Mainz, 55131 Mainz, Germany; frank.traub@unimedizin-mainz.de; 9 G.E.R.N. Center for Tissue Replacement, Regeneration & Neogenesis, Department of Orthopedics and Trauma Surgery, Medical Center—University of Freiburg, Faculty of Medicine, University of Freiburg, Engesserstr. 4, 79108 Freiburg, Germany

**Keywords:** rhabdomyosarcoma, sclerosing, spindle cell, cell line establishment, stem cell, genetics, differentiation, MYOD1, p53, WNT

## Abstract

Sclerosing spindle cell rhabdomyosarcoma (SSRMS) is a rare rhabdomyosarcomas (RMS) subtype. Especially cases bearing a myogenic differentiation 1 (*MYOD1*) mutation are characterized by a high recurrence and metastasis rate, often leading to a fatal outcome. SSRMS cell lines are valuable in vitro models for studying disease mechanisms and for the preclinical evaluation of new therapeutic approaches. In this study, a cell line established from a primary SSRMS tumor of a 24-year-old female after multimodal chemotherapeutic pretreatment has been characterized in detail, including immunohistochemistry, growth characteristics, cytogenetic analysis, mutation analysis, evaluation of stem cell marker expression, differentiation potential, and tumorigenicity in mice. The cell line which was designated SRH exhibited a complex genomic profile, including several translocations and deletions. Array-comparative genomic hybridization (CGH) revealed an overall predominating loss of gene loci. The mesenchymal tumor origin was underlined by the expression of mesenchymal markers and potential to undergo adipogenic and osteogenic differentiation. Despite myogenic marker expression, terminal myogenic differentiation was inhibited, which might be elicited by the *MYOD1* hotspot mutation. In vivo tumorigenicity could be confirmed after subcutaneous injection into NOD/SCID/γ_c_^null^ mice. Summarized, the SRH cell line is the first adult SSRMS cell line available for preclinical research on this rare RMS subtype.

## 1. Introduction

Rhabdomyosarcomas (RMS) are rare mesenchymal tumors showing a partial striated muscle cell differentiation [1]. While RMS are the most prevalent soft tissue sarcomas (STS) in children and adolescents, making up about 3% of all malignancies at that age [2], in adults liposarcomas and leiomyosarcomas are the dominating STS [3]. The worldwide age standardized RMS incidence rate is 0.3/100,000 [3]. Several distinct histopathological RMS subtypes have been defined: young children are most often affected by the embryonal RMS (ERMS) subtype, the proportion of alveolar RMS (ARMS) increases in older children and adolescents [4], whereas, in adults, the pleomorphic (PRMS) subtype is most frequently diagnosed [5].

Pediatric RMS with spindle cell histotype were first described in 1992 [6], followed by adult spindle cell RMS in 1998 [7]. In addition, a sclerosing histotype in adults was discovered in 2000 [8]. Later, the frequent coexistence of spindle cells with hyalinized sclerosing areas in the same tumor led to the definition of sclerosing spindle cell RMS (SSRMS) as an independent histopathologic RMS subtype [9]. Unlike an infantile variant of SSRMS that is characterized by the presence of nuclear receptor coactivator (NCOA) fusion proteins, which has a favorable prognosis [10,11], most SSRMS in adolescents and adults express myogenic differentiation 1 (MYOD1) and they are highly aggressive marked by frequent recurrence and metastasis [12,13].

Additionally, desmin is frequently expressed by SSRMS and a focal positivity for myogenin (MYOG), combined with a high proliferative index (marker of proliferation Ki-67), has been reported, while the ARMS typic paired box (PAX)-forkhead box protein O1 (FOXO1) fusion proteins are absent in SSRMS [14,15,16]. Though expressing myogenic marker proteins, the completion of myogenic differentiation is inhibited in SSRMS, due to frequently observed recurrent MYOD1 mutations [16,17,18,19]. 

Several chromosomal aberrations have been reported for SSRMS tumors, including amplifications of 12q13–15 coding for mouse double minute 2 (MDM2) and high mobility group AT-hook 2 (HMGA2) [20], the loss of 10q22 along with gain of chromosome 18 [21], or a complex karyotype including gain of chromosome 11 and loss of chromosome 22, which was associated with the gain of the whole chromosomes 16, 18, and 21, and a partial gain of chromosome 1 in a subset of cells [22]. 

Multimodal chemotherapy and radiation therapy are frequently applied upon the diagnosis of SSRMS. Yet, inherent and acquired therapeutic resistance have been reported for several SSRMS cases, especially for those with *MYOD1* mutations [12,23,24]. 

Stem cell features of ERMS have been explored during the recent years. Proposed cancer stem cell (CSC) markers are prominin-like protein 1 (PROM1), also known as CD133 [25], aldehyde dehydrogenase 1 (ALDH1) [26], as well as members of the ABC transporter family [26]. In addition, pluripotency markers, like SRY-box transcription factor 2 (SOX2) and c-Myc (MYC), which is also an important oncogene, were found to be concomitantly elevated with ALDH1 [26]. Specifically, for SSRMS, no stem cell marker analysis has been published so far.

The downregulation of the wingless-type (WNT) signaling concurrently with activation of hedgehog (Hh) signaling, two pathways which are involved in stemness as well as myogenic differentiation, has been reported for ERMS and ARMS [27,28]. 

To date, the establishment of only one SSRMS cell line has been published recently [29]. The cell line designated SRH that is presented in this paper has been already included in RMS research showing a distinct drug response in vitro when compared to popular ERMS and ARMS cell lines [30,31]. Here, we provide the detailed characterization of the SRH cell line, including histopathology and immunohistochemistry, growth characteristics, cytogenetic analysis, mutation analysis, evaluation of stem cell marker expression, differentiation potential, and tumorigenicity in mice. 

## 2. Materials and Methods

### 2.1. Patient History

The SRH cell line was established at the University Hospital Tuebingen from a primary tumor that was located in the left lower leg of a 24-year-old female. The initial diagnosis of a sclerosing spindle cell rhabdomyosarcoma with multiple osseous metastases was obtained six months before resection. Despite subsequent multimodal chemotherapy, including vincristine, adriamycin, ifosfamide, actinomycin-D, carboplatin and etoposide, upon resection, the progressive 23 × 11 × 12 cm large primary tumor contained >50% vital tumor cells. Twelve months after the initial diagnosis, the patient succumbed to metastatic disease affecting the lungs, skull, pelvis, spine, and right femoral neck. 

The ethics committee of the medical faculty Tuebingen, project no. 612/2010 BO2, approved the study. The patient provided written informed consent to take part in the study.

### 2.2. Histopathology and Immunohistochemistry

Routine histological staining (H&E) was performed on 3–5 µm thick sections of formalin-fixed and paraffin-embedded samples of the original SRH tumor following standard protocols. Immunohistochemistry was carried out with an automated immunostainer (Ventana Benchmark Ultra, Roche Diagnostics, Mannheim, Germany), according to the manufacturer’s instructions with the following antibodies: Desmin mouse mAb (clone D33, Agilent Technologies, Waldbronn, Germany), MyoD1 rabbit mAb (clone EP212, Roche Diagnostics, Mannheim, Germany), and Ki-67 mouse mAb (clone MIB-1, Agilent Technologies, Waldbronn, Germany). Appropriate positive and negative controls were employed in order to confirm the adequacy of the staining. The enhancement, extent, and pattern of specific antibody immunostaining within a tissue section were determined. The sections were inspected at 50×, 100×, or 400× magnification by an expert pathologist.

### 2.3. Primary Cell Culture and Propagation of SRH Cells

A portion of the resected primary tumor was washed twice in phosphate-buffered saline (PBS) (Thermo Fisher Scientific, Waltham, MA, USA), cut into small pieces of 1–2 mm^3^ and initially propagated as outgrowth culture. Tissue fragments were placed in 25 cm^2^ cell culture flasks and cultured in Dulbecco’s minimal essential medium (DMEM) containing 4.5 g/L glucose (Thermo Fisher Scientific, Waltham, MA, USA) that was supplemented with 10% heat-inactivated fetal bovine serum (Thermo Fisher Scientific, Waltham, MA, USA) and 1× antibiotic-antimycotic solution (Thermo Fisher Scientific, Waltham, MA, USA) with 100 units/mL penicillin, 100 µg/mL streptomycin, and 0.25 µg/mL amphothericin B. The cells were maintained at 37 °C in a humidified 5% CO_2_ atmosphere.

An exchange of culture medium was performed twice weekly until stable cell growth was established. Subsequently, cells were subcultured once a week by 0.025% trypsin/EDTA (Thermo Fisher Scientific, Waltham, MA, USA) dissociation. During the subsequent period of continuous propagation, the cells were cryopreserved in 90% FBS and 10% DMSO (Merck, Darmstadt, Germany) and then stored in liquid nitrogen. Active culturing of the SRH cell line was performed until passage 75 without changes in the growth rate or morphology. For all experiments, SRH cells of passage 5–10 were used. 

The SRH cell line was regularly tested for the absence of mycoplasma contamination while using the PCR Mycoplasma Test Kit I/C (PromoCell, Heidelberg, Germany).

Phase contrast images were taken with an Olympus IX50 inverted microscope (Olympus, Tokyo, Japan) that was equipped with a Canon EOS 200D camera (Canon, Tokyo, Japan).

### 2.4. Routine Cell Culture

Bone marrow-derived mesenchymal stem cells (MSC) were isolated at the University Hospital Tuebingen after written informed consent of the patients (approved by the ethics committee of the medical faculty, project no. 401/2013 BO2), propagated, as previously described [32], and confirmed to represent multi-lineage differentiation potential toward chondrocytes, adipocytes, and osteocytes (data not shown).

Human skeletal muscle cells (SKMC) were purchased from PromoCell (Heidelberg, Germany) and then cultured in Dulbecco’s Modified Eagle Medium (DMEM) containing 2 mM GlutaMAX, 4.5 g/L glucose, 1 mM sodium pyruvate (Thermo Fisher Scientific, Waltham, MA, USA) supplemented with 10% heat-inactivated fetal bovine serum (Thermo Fisher Scientific, Waltham, MA, USA), and 1× antibiotic-antimycotic solution (Thermo Fisher Scientific, Waltham, MA, USA).

All of the cells were cultivated at 37 °C in a humidified atmosphere containing 5% CO_2_ and were regularly tested for the absence of mycoplasma contamination while using the PCR Mycoplasma Test Kit I/C (PromoCell, Heidelberg, Germany).

### 2.5. Ultrastructure Analysis

For electron microscopy, SRH cells were trypsinized and then washed twice with pre-warmed PBS. After centrifugation, the cell pellet was fixed with Karnovsky fixative (3% paraformaldehyde and 3.6% glutaraldehyde in 0.1 M sodium cacodylate buffer, Merck, Darmstadt, Germany) for 30 min. at room temperature and stored at 4 °C. Post-fixation was based on 1.0% osmium tetroxide containing 1.5% potassium ferrocyanide for 2 h. According to standard methods, the blocks were embedded in glycidyl ether and cut while using an ultramicrotome (Ultracut, Reichert, Vienna, Austria). Ultra-thin sections (30 nm) were mounted on copper grids and analyzed using a Zeiss LIBRA 120 transmission electron microscope (Zeiss, Oberkochen, Germany) operating at 120 kV.

### 2.6. Proliferation Kinetics

The SRH cells were seeded into a 96-well plate at densities ranging from 2500 cells to 312 cells per well in two-fold dilutions (six wells each) and then cultivated for 24 h before starting bright field image acquisition and data analysis. The cells were counted daily over a period of seven days using the Celigo^®^ S Imaging Cytometer (Nexcelom Bioscience, Lawrence, MA, USA). A growth curve was plotted by algorithms of the raw data images and cell population doubling time was calculated.

### 2.7. Cell Cycle Analysis

The SRH cells were seeded into a 48-well plate at a density of 1 × 10^4^ cells per well and they were cultivated for 24 h. The proliferating cells were labeled with 10 µM EdU for 4 h, harvested and stained according to the protocol for the Click-iT™ EdU Alexa Fluor 647 Flow Cytometry Assay Kit (Thermo Fisher Scientific, Waltham, MA, USA). FxCycle™ PI/RNase Staining Solution (Thermo Fisher Scientific, Waltham, MA, USA) was used for the staining of cellular DNA before analyzing the cells by flow cytometry (BD FACSCanto™ II Cell Analyzer, BD Biotechnologies) for cell cycle distribution.

### 2.8. Multicolor Fluorescence In Situ Hybridization (mFISH) and Chromosomal Breakpoint Analysis

Multicolor fluorescence in situ hybridization (mFISH) analysis was carried out on metaphase slides while using a human chromosome-specific mFISH kit (MetaSystems, Altlussheim, Germany). The mFISH procedure was performed according to the manufacturers’ instructions and as previously described [33]. Fluorochromes were sequentially captured using specific single-band pass filters in a Zeiss Axioplan 2 microscope (Zeiss, Oberkochen, Germany). mFISH ISIS software (MetaSystems, Altlussheim, Germany) was used for image analysis. At least five metaphases were analyzed. The karyotype was described according to the International System of Human Cytogenetic Nomenclature (ISCN) 2016. Chromosomal breakpoints were determined by G-banding analysis while using a standard protocol according to the ISCN nomenclature. The CyDAS software package (http://www.cydas.org/OnlineAnalysis/) was used to extract chromosomal gains and losses as well as breakpoints from the karyotype.

### 2.9. Array-Comparative Genomic Hybridization (CGH) Analysis

Array-based comparative genomic hybridization (array-CGH) was performed by Miltenyi Biotec (Bergisch Gladbach, Germany) while using an Agilent Human Genome CGH Microarray 244K (Agilent Technologies, Waldbronn, Germany) consisting of 244,000 in situ synthesized 60-mer oligonucleotides spanning the entire human genome with an average probe spacing of 6.5 kb.

Genomic DNA of the SRH cell line and a female reference DNA (Promega, Mannheim, Germany) for control were labeled with Cy5-dCTP and Cy3-dCTP, respectively, and then hybridized to an oligonucleotide microarray, according to the Agilent oligonucleotide array-based CGH for genomic DNA analysis protocol v5.0 while using the Agilent Oligo aCGH Hybridization Kit. The fluorescence signals of the hybridized microarray were detected using Agilent’s DNA microarray scanner. The Agilent Feature Extraction software was used in order to read out and process the microarray image file. Further analysis and the visualization of the hybridization result were performed with the Agilent CGH Analytics software v3.4 with the following aberration filter settings: a minimum number of probes present in an aberrant region = 2; minimum absolute average log2 ratio for region = 0.4 corresponding to a-fold change of 1.32; and, the ADM-2 algorithm was used for statistical analysis.

### 2.10. Ingenuity Pathway Analysis

Ingenuity pathway analysis (IPA, Qiagen, Hilden, Germany) was performed in order to determine the possible interactions of gene products that are located in chromosomal regions with gains or losses, as identified by array-CGH, according to the manufacturer’s instructions.

### 2.11. DNA Preparation

Genomic DNA was isolated from the original primary tumor tissue, the established SRH cell line, and tumor xenografts while using the QiaAmp DNA Blood Mini Kit (Qiagen, Hilden, Germany). A NanoDrop™ 2000 microvolume spectrophotometer (Thermo Fisher Scientific, Waltham, MA, USA) was used in order to quantify and assess purity of DNA.

### 2.12. Mutation Analysis

Mutation analysis was performed for *TP53* (exons 4–9) and *MYOD1* (exon 1). Primers for the detection of the MYOD1 p.L122R hot spot mutation were used according to Agaram et al. [17]. The primers for *TP53* were used according to Das et al. [34]. The direct sequencing of DNA amplicons was performed on an ABI 3130xl Genetic Analyzer while using a BigDye Terminator v3.1 Cycle Sequencing Kit (Thermo Fisher Scientific, Waltham, MA, USA). The sequences were analyzed using Chromas-Pro v2.6.6 software (Technelysium Pty Ltd., South Brisbane, Australia) and verified against sequences of human *TP53* and *MYOD1* deposited at the National Center for Biotechnology Information (NCBI) USA for reference.

### 2.13. RNA Isolation and Quantitative Real Time PCR

The total RNA was extracted from the newly established SRH cell line and human SKMC while using the RNeasy Plus Kit (Qiagen). The purity and concentration of RNA was analyzed with a NanoDrop™ 2000 microvolume spectrophotometer (Thermo Fisher Scientific, Waltham, MA, USA). Random-primed cDNA was synthesized from 1.0 µg of total RNA while using the SuperScript™ VILO™ cDNA Synthesis Kit (Thermo Fisher Scientific, Waltham, MA, USA), according to the manufacturer’s instructions. 

The following qRT-PCR primers were used, as previously published: fatty acid binding protein 4 (*FABP4*) Köllmer et al. [35] and osteopontin (*OPN*) Wang-Rodriguez et al. [36].

Additional primer pairs that were purchased from Eurofins Genomics (Ebersberg, Germany, see Table 1) were designed with the Primer-BLAST software (National Center for Biotechnology Information, NCBI), employing common design parameters. The specificity of the amplicons was checked in silico while using the BLAT (UCSC Genome Browser) and BLAST (NCBI) alignment tools.

Gene expression analysis was performed by quantitative real time PCR (qRT-PCR) while using gene-specific primers and the SYBR Select Master Mix for CFX (Thermo Fisher Scientific, Waltham, MA, USA) on a CFX96 Real-Time PCR Detection System (Bio-Rad Laboratories, Hercules, CA, USA). For data analysis, the CFX Maestro™ software v2.0 (Bio-Rad Laboratories, Hercules, CA, USA) was used and the relative expression levels were calculated with the 2^−ΔΔCt^ method according to Livak et al. 2001 [37], with TATA box binding protein (*TBP*) or tyrosine 3-monooxygenase/tryptophan 5-monooxygenase activation protein zeta (*YWHAZ*) as a reference gene [38]. 

### 2.14. Flow Cytometry Analysis of Cell Surface Markers

The cells were detached while using Accutase^®^ (Stemcell Technologies, Vancouver, BC, Canada), washed twice in PBS, and then stained with fluorochrome-conjugated antibodies (using the antibody concentrations recommended by the manufacturer) in FACS buffer that is composed of PBS supplemented with 2% FBS and 2 mM EDTA for 15 min. at 4 °C. After centrifugation at 300× *g* for 5 min. at 4 °C, the cells were washed twice with cold FACS buffer before being subjected to flow cytometry. Cell analysis was performed on a BD FACSCanto™ II (BD Biosciences) flow cytometer while using BD FACSDIVA™ software v8.0.1 (BD Biosciences, Franklin Lakes, NJ, USA). FlowJo™ software v10.0.8 (BD Biosciences, Franklin Lakes, NJ, USA) was used for data analysis.

Antibodies against cell surface markers reflecting distinct cell fates and stages in the mesenchymal and/or hematopoietic lineage were used for cell labeling: CD10-PE (clone HI10a, BD Biosciences, Franklin Lakes, NJ, USA), CD14-PE (clone U52E, BD Biosciences, Franklin Lakes, NJ, USA), CD19-APC (clone HIB19, BioLegend, San Diego, CA, USA), CD34-APC (clone 581, BioLegend, San Diego, CA, USA), CD44-APC (clone BJ18, BioLegend, San Diego, CA, USA), CD45-APC (clone 5B1, Miltenyi Biotech, Bergisch-Gladbach, Germany), CD73-APC (clone AD2, BioLegend, San Diego, CA, USA), CD90-PE (clone 5E10, BioLegend, San Diego, CA, USA), CD105-PE (clone 5N6, eBioscience, San Diego, CA, USA), CD146-PE (clone P1H12, BioLegend, San Diego, CA, USA), and CD166-PE (clone 3A6, eBioscience, San Diego, CA, USA). Matching isotype controls were included in each experiment.

### 2.15. Tumor Sphere Formation Assay

SRH single cell suspensions that were generated by trypsin-EDTA dissociation were prepared for sphere formation assays. The cells were seeded in NeuroCult™ NS-A Proliferation Medium (Stemcell Technologies, Vancouver, BC, Canada), supplemented with 20 ng/mL recombinant human EGF (Miltenyi Biotech Bergisch Gladbach, Germany), 10 ng/mL recombinant human bFGF (Miltenyi Biotech Bergisch Gladbach, Germany), and 2 µg/mL heparin (Stemcell Technologies, Vancouver, BC, Canada) at a density of 10–1000 cells/well in Nunclon Sphera™ ultra-low attachment flat bottom 96-well plates (Thermo Fisher Scientific, Waltham, MA, USA). SRH spheres were cultured at 37 °C in humidified air containing 5% CO_2_ for seven days before being scored with the Celigo^®^ S Imaging Cytometer (Nexcelom Bioscience, Lawrence, MA, USA). The sphere forming efficiency (%) was calculated as the number of tumor spheres divided by number of cells seeded × 100.

### 2.16. Osteogenic and Adipogenic Differentiation

SRH cells were maintained in osteogenic and adipogenic differentiation media for 21 days. The osteogenic induction medium consisted of DMEM high glucose (4.5 g/L), supplemented with 10% fetal bovine serum, 0.1 μM dexamethasone, 10 mM β-glycerol phosphate, and 50 μM L-ascorbic acid (Merck, Darmstadt, Germany). Adipogenic differentiation medium was purchased from PromoCell (Heidelberg, Germany). Alizarin Red S (Carl Roth, Karlsruhe, Germany) and Oil Red O (Thermo Fisher Scientific, Waltham, MA, USA) were utilized according to the manufacturer’s instructions to depict osteogenic and adipogenic differentiation, respectively. 

Intracellular lipid accumulation after adipogenic differentiation was detected by staining with the HCS LipidTOX™ Green neutral lipid stain (Thermo Fisher Scientific, Waltham, MA, USA), according to the manufacturer’s instructions. The cells were mounted in VectaShield^®^ HardSet™ antifade mounting medium with 4′,6-diamidino-2-phenylindole (DAPI) (Vector Laboratories, Burlingame, CA, USA), before being visualized while using an Axio Imager Z1 phase contrast fluorescence microscope (Zeiss, Oberkochen, Germany) that was equipped with an AxioCam MRm camera (Zeiss, Oberkochen, Germany) and AxioVision software v4.8 (Zeiss, Oberkochen, Germany). Cell differentiation and staining was performed in three independent experiments with a representative image shown.

### 2.17. Tumorigenicity in Mice

Animal experiments were performed with the authorization of the Institutional Animal Care and Use Committee of the University of Tuebingen, according to German federal and state regulations (approval no. M04/09). Female NOD/SCID/γ_c_^null^ (NSG) mice (*n* = 3), six weeks of age, were subcutaneously inoculated with 5 × 10^6^ SRH tumor cells in cold Matrigel™ (BD Biosciences, Franklin Lakes, NJ, USA). The mice were sacrificed after 8–10 weeks when the tumors had reached a maximum volume of 1000 mm^3^.

### 2.18. Assessment of Cell Line Identity

DNA profiling was performed for the verification of human cell line identity between the original primary tumor tissue from the patient, the established SRH cell line, and a tumor xenograft of SRH cells that were propagated in NOD/SCID/γ_c_^null^ mice while using the StemElite™ ID System (Promega, Mannheim, Germany). DNA was extracted and amplified according to the manufacturer’s instructions. The StemElite™ ID System uses short tandem repeat (STR) analysis of specific loci in the human genome through co-amplification and three-color detection of ten loci: nine autosomal loci (D21S11, TH01, TPOX, vWA, CSF1P0, D16S539, D7S820, D13S317, D5S818) and amelogenin (AMEL) for gender identification. Additionally, the StemElite™ ID System includes a sensitive marker that specifically detects the presence of DNA from mouse (*Mus musculus*). Electrophoretic analysis was carried out while using a performance optimized polymer (POP-4TM) with an ABI 3130xl Genetic Analyzer (Applied Biosystems, Foster City, CA, USA). Each electrophoretic run was analyzed with the GeneMapper v4.0 software (Applied Biosystems, Waltham, MA, USA) and then compared with the StemElite™ ID allelic ladders.

The resulting STR profiles were compared with cell lines in public databases, including the American Type Tissue Culture Collection (ATCC), the Children’s Oncology Group (COG) Cell Culture and Xenograft Repository, the Deutsche Sammlung von Mikroorganismen und Zellkulturen (DMSZ), and the Japanese Collection of Research Bioresources (JCRB) while using the Cell Line Integrated Molecular Authentication Database (CLIMA) 2.1.

## 3. Results

### 3.1. Histopathology and Immunohistochemistry of the Original Tumor

**Figure 1:**
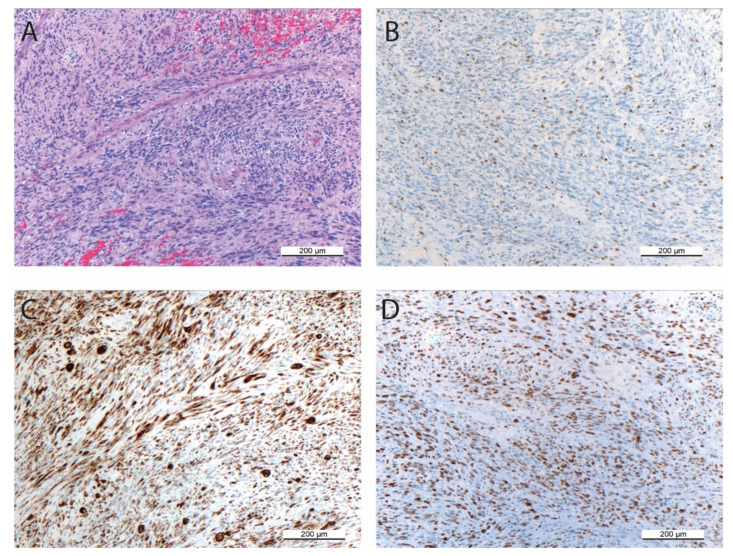
Histologic examination of the primary tumor from which the SRH cell line was established. (**A**) H&E staining (100× magnification) showing the spindle cell morphology with scattered larger tumor cells mainly organized in fascicular patterns. (**B**) KI-67 positivity indicating proliferation activity was analyzed by immunohistochemistry (100× magnification). (**C**) Desmin expression was determined by immunohistochemistry (100× magnification). (**D**) Nuclear MYOD1 reactivity was evaluated by immunohistochemistry (100× magnification).

The primary tumor from which the SRH cell line was established predominantly consisted of spindle cell parts with scattered larger tumor cells in a sclerosing matrix (Figure 1A).

Despite multimodal chemotherapeutic pretreatment, up to five mitoses were detectable per HPF. The proliferation activity was also confirmed by Ki-67 positivity (Figure 1B) in 10–20% of cells. Desmin and MYOD1 positivity (Figure 1C,D) of most cells, irrespective of their morphology, indicated the myogenic origin of the tumor.

### 3.2. Morphology of the SRH Cell Line and Growth Characteristics

**Figure 2:**
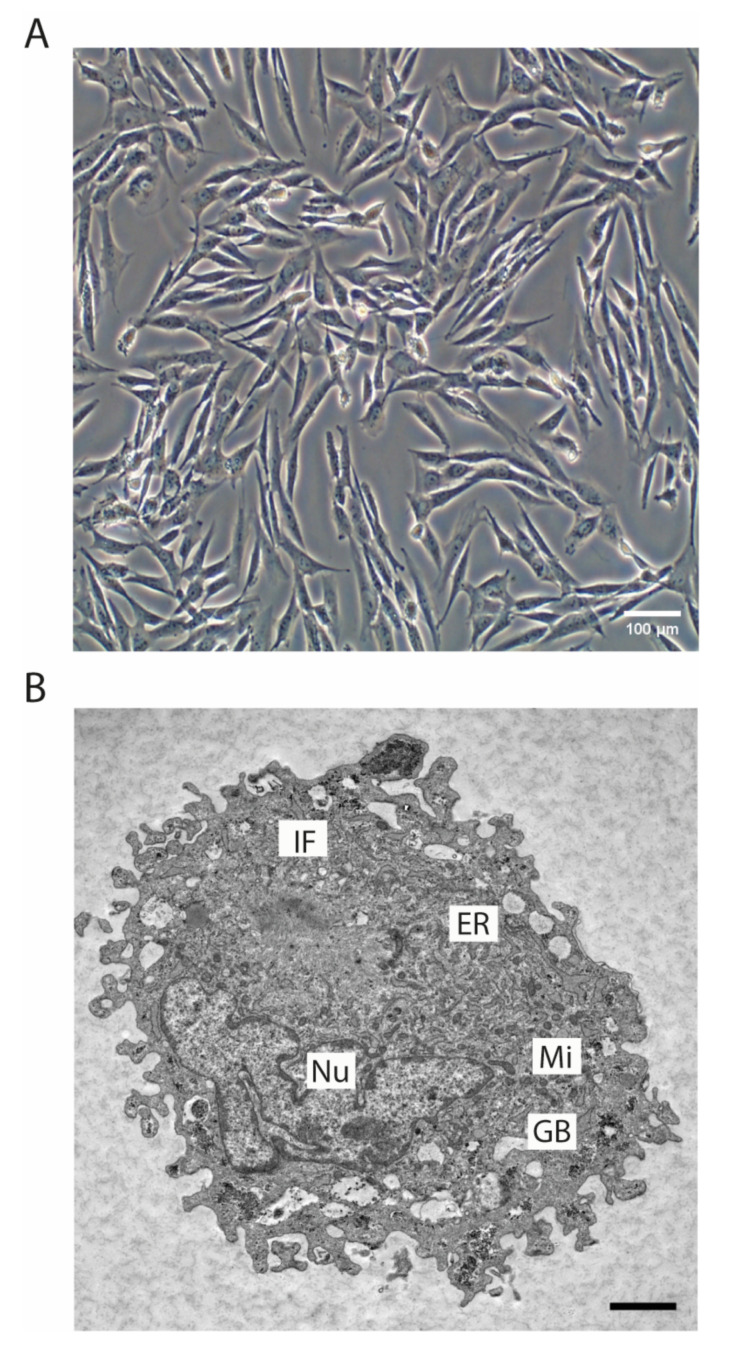
SRH cell line phenotype. (**A**) Phase contrast image of SRH cells in monolayer culture (20× magnification). (**B**) Transmission electron microscope (TEM) image of a representative SRH cell: Nucleus (Nu), mitochondria (Mi), endoplasmic reticulum (ER), glycogen bodies (GB), intermediate filaments (IF) (scale bar 2 µm).

The SRH cell line was established as outgrowth culture of the original primary tumor. The phase contrast image (Figure 2A) showed the spindle cell morphology of the SRH cell line that was propagated as monolayer culture.

The TEM micrograph revealed an elongated, irregular shaped nucleus. Glycogen bodies that were indicating glycogen storage were present in SRH cells. In addition, intermediate filaments resembling a partial striated muscle cell differentiation could be detected (Figure 2B).

**Figure 3:**
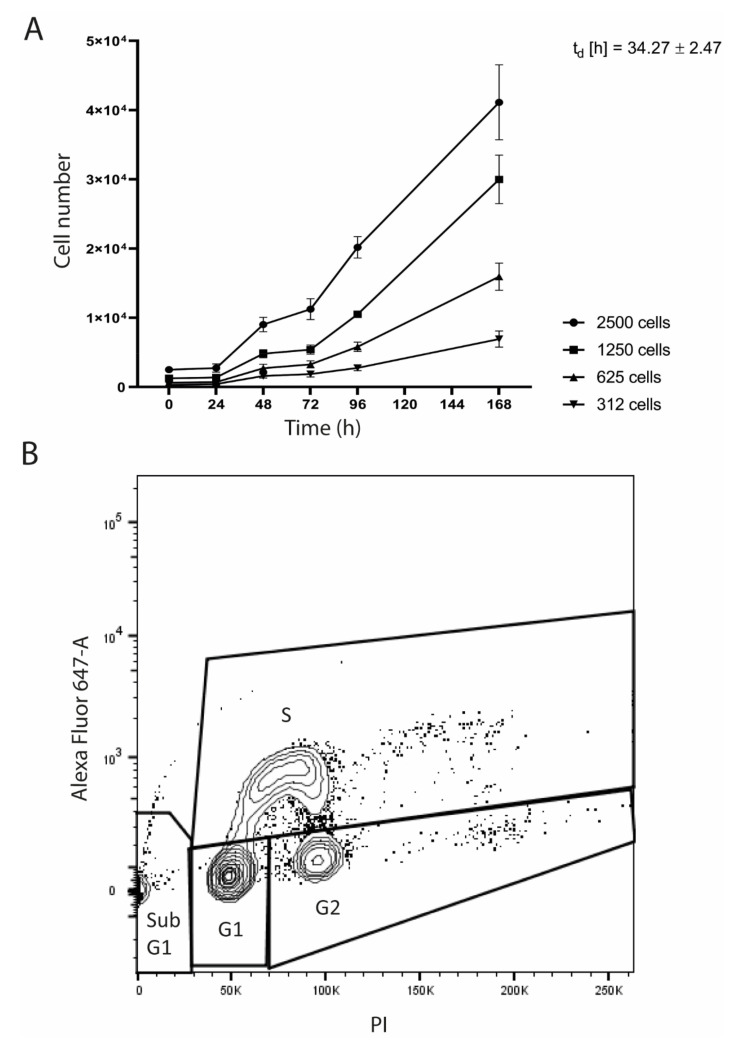
SRH proliferation characteristics. (**A**) Growth curves were determined over a period of 168 h (h) after seeding of 312, 625, 1250 or 2500 SRH cells and the cell population doubling time (t_d_) was calculated. (**B**) Cell cycle (EdU Alexa Fluor 647-A) and DNA ploidy (PI) analysis was performed by flow cytometry. Distribution of the cells in the different cell cycle phases sub G1, G1, G2, and S was analyzed.

The growth curves revealed a continuous growth with a cell population doubling time (t_d_) of 34.3 ± 2.5 h (Figure 3A).

Flow cytometry showed the cell cycle distribution of SRH cells with 1.6% in sub G1, 70.0% in G1, 10.3% in G2, and 18.1% in S phase (Figure 3B).

### 3.3. Cytogenetic Analysis by mFISH Karyotyping and Chromosomal Breakpoint Analysis

**Figure 4:**
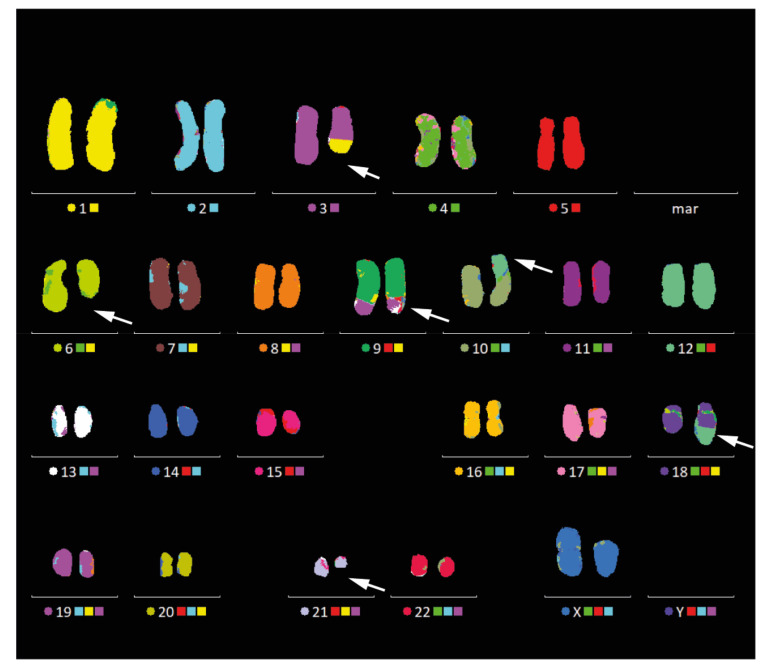
Multi-color fluorescence in situ hybridization (mFISH). 24-color karyotyping of the SRH cell line revealed complex genomic aberrations. White arrows indicate the derivative chromosomes.

Karyotype analysis of SRH cells while using G-banding and mFISH analysis revealed complex genomic aberrations (Figure 4), yet, without a gain or loss of whole chromosomes: 46, XX, t(1;3;9) (p34; p14;q34), del (3) (q24), del (6) (q14q24), +der (9) t (1;3;9) add (9) (p23), der (10) t (10;12) (p11; q13), der (18) t (12;18) (q13;q22), and del (21) (q21q22).

Parts of chromosome 12 were translocated to chromosome 10 and 18, leading to a tetraploidy of the long arm of chromosome 12 (12q13-qter). This was confirmed by array-CGH, which revealed gains of chromosome 12 p13, p11, q13–q15, and q21–q24. The CyDAS data analysis system was used in order to analyze genomic aberrations encoded by the karyotype and the detailed results are listed below:

Break Points: 1p34,3p14,3q24,6q14,6q24,9p23,9q34,10p11,12q13,18q22,21q21,21q22

Structural: t(1)(p34), t(3)(p14), del(3)(q24), del(6)(q14), del(6)(q24), add(9)(p23), t(9)(q34), t(10)(p11), t(12)(q13), t(18)(q22), del(21)(q21), del(21)(q22)

Structural at 400bphs: t(1)(p343p341), t(3)(p14), del(3)(q24), del(6)(q14), del(6)(q24), add(9)(p23), t(9)(q34), t(10)(p112p111), t(12)(q13), t(18)(q22), del(21)(q21), del(21)(q22)

Quantitative: +(3)(pterp14), −(3)(q24qter), −(6)(q14q24), +(9)(p23q34), −(10)(pterp11), +(12)(q13qter)×2, −(18)(q22qter), −(21)(q21q22), +(?)(??)

Quantitative at 400bphs: +(3)(p26p14), −(3)(q24q29), −(6)(q14q24), +(9)(p23q34), (10)(p15p111), +(12)(q13q243)×2, −(18)(q22q23), −(21)(q21q22)

Junctions: (+)(1)(1p34)::(3)(3p14)(+), (+)(1)(1p34)::(9)(9q34)(+), (+)(3)(3p14)::(9)(9q34)(+)×2, (+)(6)(6q14)::(6)(6q24)(−), (−)(9)(9p23)::(?)(?)(?), (−)(10)(10p11)::(12)(12q13)(−), (−)(12)(12q13)::(18)(18q22)(+), (+)(21)(21q21)::(21)(21q22)(−)

CKAS Ploidy/Numerical/Balanced/Unbalanced/Unclassified/Overall): 0/1/4/4/0/9

### 3.4. Array-CGH and Pathway Analysis

Cytogenetic characterization of SRH cells was performed by array-CGH (Supplementary Figure S1 and Supplementary Table S1). The SRH cells were characterized by a complex molecular karyotype with many gains and losses in cytogenetic regions containing candidate genes for pathways that are involved in stemness and tumorigenesis. Overall, more chromosomal regions with losses could be identified. Only for chromosome 12 gains were prevailing, which covered p13, p11, q13–q15, and q21–q24. Any discrepancies between mFISH karyotyping and array-CGH are due to the lower sensitivity of G-banding analysis.

**Figure 5:**
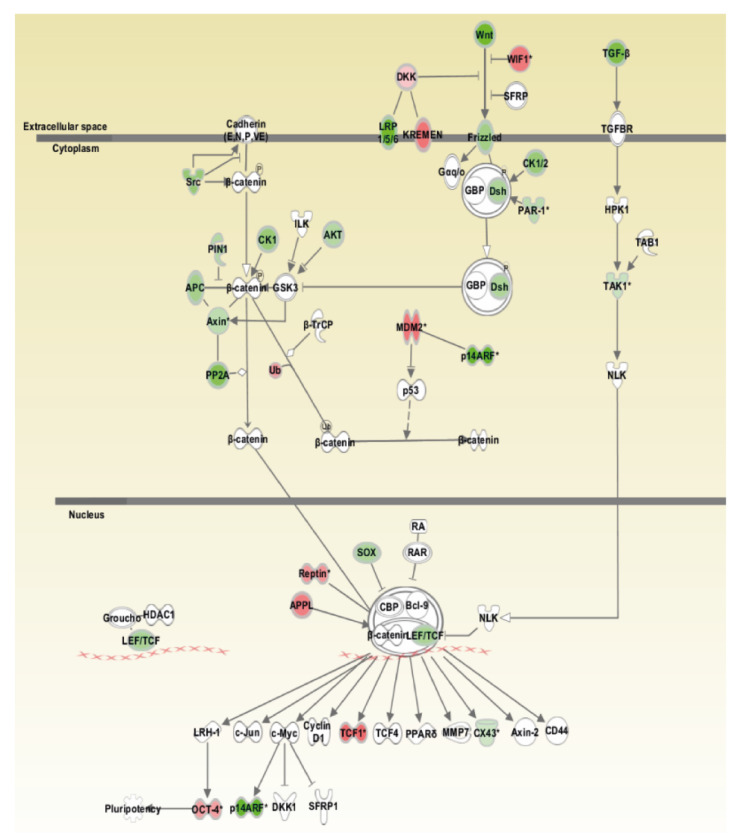
Wingless-type (WNT) signaling pathway. To investigate possible interactions of genes located in chromosomal regions with gains or losses, as determined by array-comparative genomic hybridization (CGH), ingenuity pathway analysis (IPA) has been performed. One of the most highly rated networks in IPA analysis was the WNT signaling pathway. The chromosomal regions coding for genes that are shaded were determined to be significant from the statistical analysis. The genes shaded red are located in chromosomal regions with gains and those that are green are located in chromosomal regions with losses. White shaded genes relate to the pathway but were not found to be altered in the actual analysis. The intensity of the shading shows to what degree an amplification or loss of the specific chromosomal region was detected. A solid line represents a direct interaction between the two gene products, arrows show activating interactions, bar-headed lines inactivation.

Figure 5 summarizes amplifications and losses of chromosomal regions coding for components of the WNT pathway.

Losses were dominating in chromosomal regions coding for components of the WNT pathway, including WNT ligands (*WNT3A*, *WNT9A*, and *WNT9B*), frizzled (*FZD*) receptor 10, dishevelled (*DSH*) 1, adenomatous polyposis coli (*APC*) 2, axis inhibition protein 1 (*AXIN*) 1, *AKT1* kinase, casein kinase 1 δ (*CSNK1D*), and members of the lymphoid enhancer binding factor/transcription factor (LEF/TCF) family (*TCF3*, *TCF21*). Gains were observed for chromosomal regions coding for the inhibitory WNT pathway components WNT inhibitory factor 1 (*WIF1*), dickkopf-like 1 (*DKKL1*), and kringle-coding gene marking the eye and the nose 1 (*KREMEN1*). Interestingly, there was also a gain of the chromosomal region coding for *TCF1*. In addition, the pluripotency gene POU domain transcription factor (*POU5F1*) and ubiquitin ligase *MDM2* gene were located in chromosomal regions with gains. 

Regarding the Hedgehog (Hh) pathway (Supplementary Figure S2), specifically gains of chromosomal regions coding for Fused homolog (*STK36*) and glioma-associated oncogene family zinc finger 1 (*GLI 1*) genes were observed.

Moreover, the Supplementary Figure S3 summarizes changes in chromosomal regions coding for components of the NOTCH pathway. Genomic regions containing the notch homolog 1 (*NOTCH1*) and hairy and enhancer of split 7 (*HES7*) genes both showed losses.

### 3.5. MYOD1 Mutation and p53 Mutations in SRH Cells

**Figure 6:**
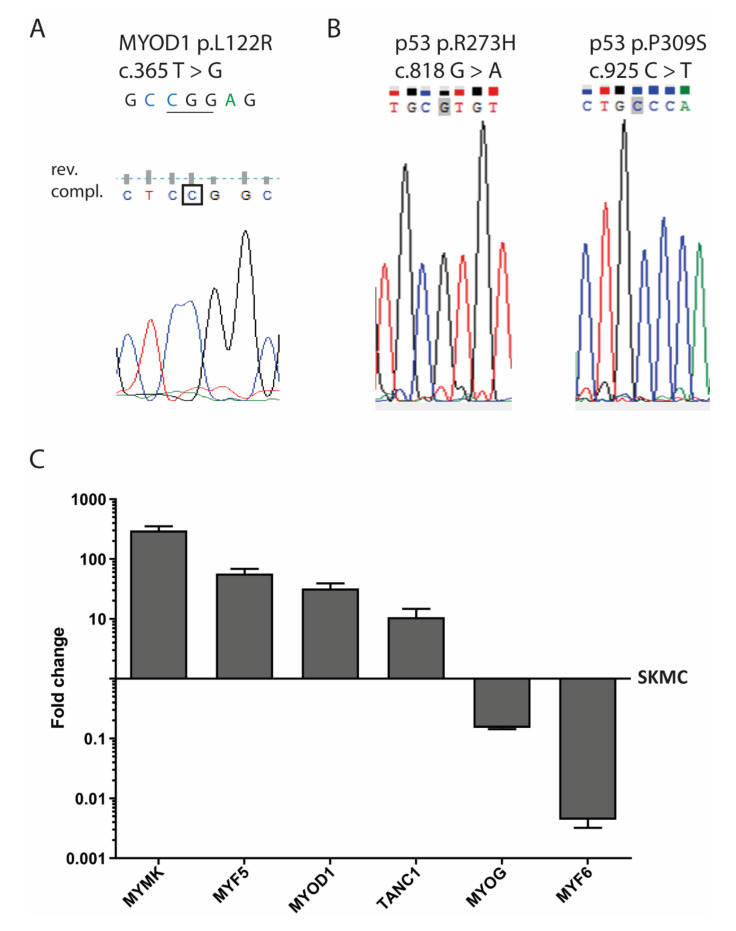
Mutation analysis and myogenic marker gene expression. Sanger sequencing of SRH cell genomic DNA showed mutations in the *MYOD1* and *TP53* gene (**A**) A *MYOD1* c.365 T > G mutation was identified. For sequencing of *MYOD1* a reverse primer was used. Therefore, the chromatogram shows the reverse complement sequence (**B**) Two *TP53* mutations at c.818 G > A and c.925 C > T were detected. (**C**) The total RNA was extracted from the SRH cell line and human SKMC. Quantitative RT-PCR was performed for the genes *MYMK*, *MYF5*, *MYOD1*, *TANC1*, *MYOG*, and *MYF6* in triplicate and normalized to the housekeeping gene *TBP*. Expression levels relative to SKMC are shown. Error bars indicate the standard deviation.

The genomic DNA of SRH cells was analyzed for mutations in *MYOD1* exon 1 (Figure 6A).

The hotspot mutation c.T365G (p.L122R) was present in a homozygous pattern. In addition, two *TP53* missense mutations c.G818A (p.R273H) and c.C925T (p.P309S) were present in the genomic DNA of the SRH cells (Figure 6B).

### 3.6. Myogenic Marker Gene Expression

When compared with human SKMC, mRNA expression of the myogenic marker myomaker (*MYMK*) (300.3 ± 52.1-fold), myogenic factor 5 (*MYF5*) (57.1 ± 11.5-fold), *MYOD1* (32.2 ± 7.2-fold), and tetratricopeptide repeat, ankyrin repeat and coiled-coil containing 1 (*TANC1*) (10.7 ± 4.0-fold) was enhanced in SRH cells (Figure 6C). Only *MYOG* (0.2 ± 0.0-fold) and especially myogenic factor 6 (*MYF6*) (0.004 ± 0.001-fold) showed a reduced mRNA expression in SRH cells when compared to SKMC. Interestingly, according to array-CGH (Supplementary Figure S1 and Supplementary Table S1), both *MYF5* and *MYF6* were located in chromosomal regions with gains.

### 3.7. Stem Cell Features of SRH Cells

**Figure 7:**
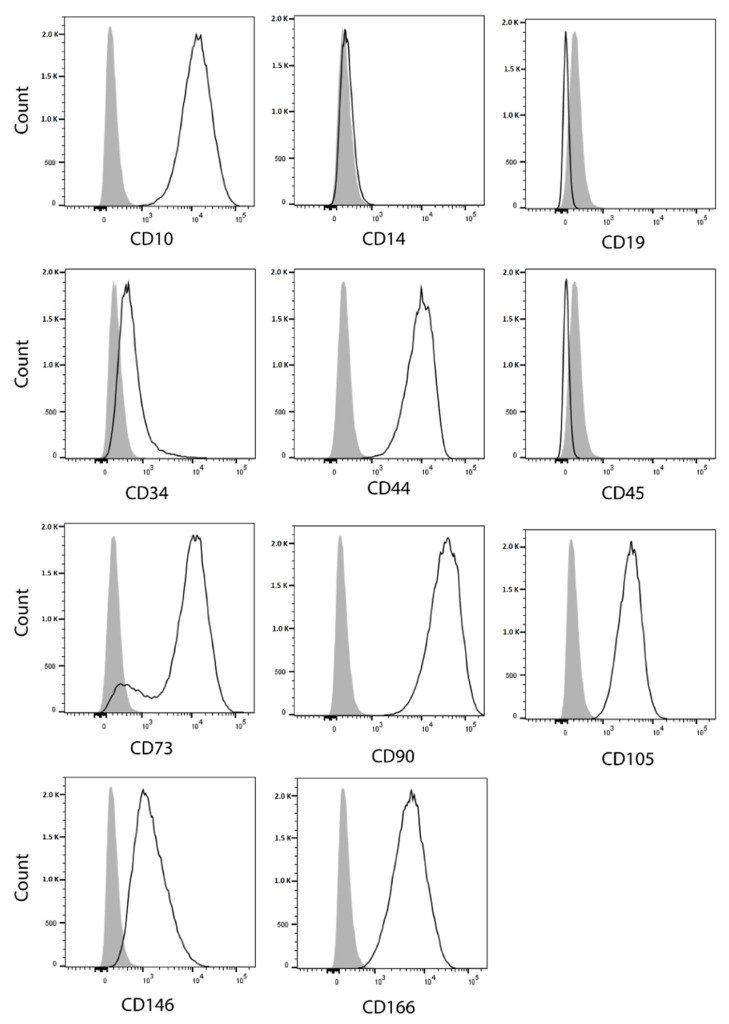
Expression of mesenchymal and hematopoietic lineage markers. Flow cytometry analysis was performed forthe cell surface markers CD10, CD14, CD19, CD34, CD44, CD45, CD73, CD90, CD105, CD146, and CD166.Open histograms (solid lines) represent specific staining profiles for the indicated antigens, whereas shaded histograms show staining with matching isotype control antibodies.

Most SRH cells that were analyzed by flow cytometry (Figure 7) were positive for surface expression of the mesenchymal marker CD10 (99.9%), CD44 (99.8%), CD73 (90.1%), CD90 (100.0%), CD105 (99.8%), CD146 (80.7%), and CD166 (99.6%), whereas no expression of the hematopoietic marker CD14, CD19, and CD45 was found.

Some SRH cells were positive for CD34 (18.6%). Yet, the median fluorescence intensity (MFI) for CD34 was only 2.2, which indicated a rather low antigen expression.

**Figure 8:**
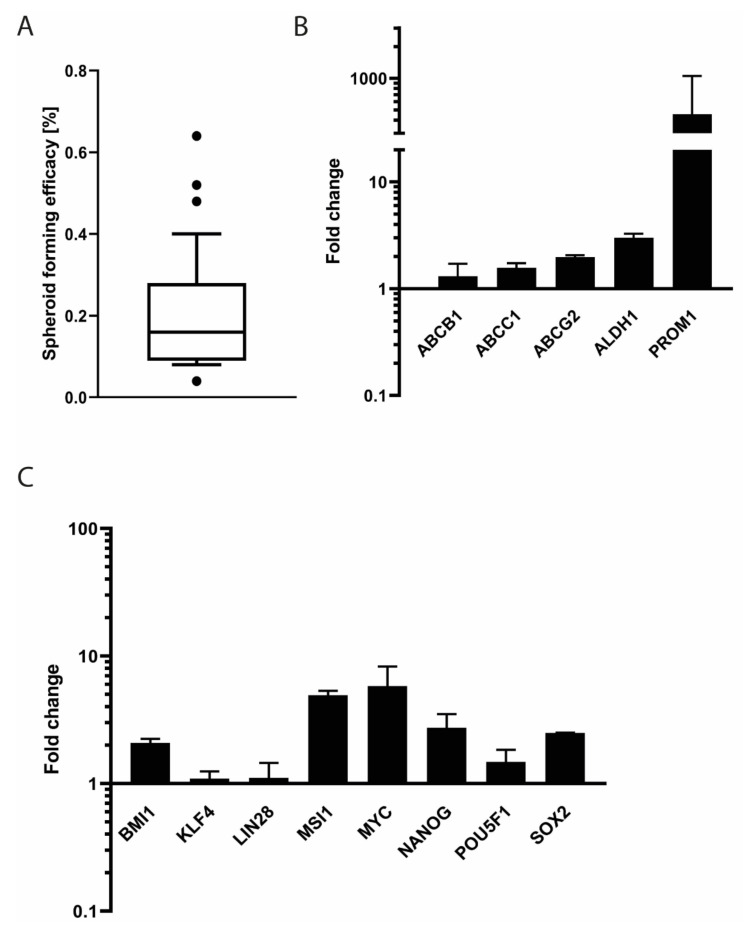
Expression of CSC and ESC markers in SRH spheres. (**A**) Sphere forming efficacy (%) was calculated as number of tumor spheres divided by number of cells seeded × 100. Outliers are indicated as dots. (**B**,**C**). Total RNA was extracted from SRH tumor spheres and monolayer cultures. Quantitative RT-PCR was performed for the genes *ABCB1*, *ABCC1*, *ABCG2*, *ALDH1*, *PROM1*, *BMI1*, *KLF4*, *LIN28*, *MSI1*, *MYC*, *NANOG*, *POU5F1*, and *SOX2* in triplicate and normalized to the housekeeping gene *YWHAZ*. The expression levels relative to monolayer culture are shown. Error bars indicate the standard deviation.

In low attachment plates, SRH cells formed loosely attached spheroids with a mean efficacy of 0.2 ± 0.1% (Figure 8A).

When compared to the monolayer culture, the SRH spheroids showed a slightly enhanced mRNA expression (Figure 8B) of the multidrug transporter ATP binding cassette subfamily B member 1 (*ABCB1*), ATP binding cassette subfamily C member 1 (*ABCC1*) and ATP binding cassette subfamily G member 2 (*ABCG2*) (1.3 ± 0.4, 1.6 ± 0.2, and 2.0 ± 0.1-fold, respectively). Additionally, the mRNA of the CSC marker *ALDH1* was 3.0 ± 0.3-fold higher expressed in spheroids as compared to monolayer cultures. The highest 453.8 ± 597.3-fold increase of mRNA expression in three-dimensional (3D) culture as compared to two-dimensional (2D) culture was found for the CSC marker *PROM1,* also known as CD133. The embryonic stem cell (ESC) marker (Figure 8C) lymphoma Mo-MLV insertion region 1 (*BMI1*) was expressed 2.1± 0.2-fold when compared to monolayer culture. Almost no differential mRNA expression compared with the 2D culture was observed for the ESC transcription factor kruppel like factor 4 (*KLF4*) (1.1 ± 0.2-fold) and the RNA binding protein lin-28 homolog A (*LIN28*) (1.1 ± 0.3-fold). The musashi RNA binding protein 1 (*MSI1*) mRNA was expressed 4.9 ± 0.4-fold higher in the spheroids when compared to control. The expression of the mRNA of the proto-oncogene *MYC* and the pluripotency factors *POU5F1*, homeobox transcription factor nanog (*NANOG*), and *SOX2* was increased 5.8 ± 2.5, 1.5 ± 0.4, 2.7 ± 0.8, and 2.5 ± 0.0-fold, respectively. 

### 3.8. Adipogenic and Osteogenic Differentiation

**Figure 9:**
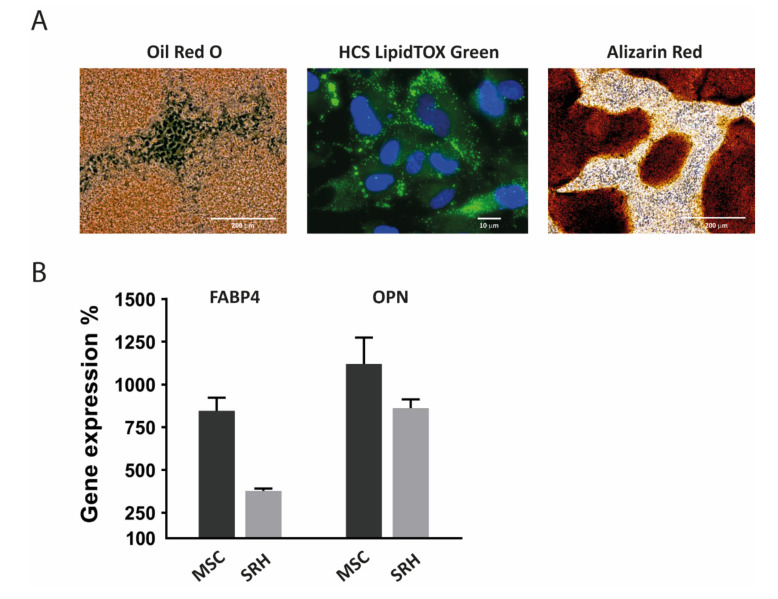
Adipogenic and osteogenic differentiation. (**A**) After 21 days of adipogenic differentiation) or osteogenic differentiation SRH cells were examined for lipid droplet formation (Oil Red O and HCS LipidTOX™ Green staining, Thermo Fisher Scientific, Waltham, MA, USA) or calcium deposition (Alizarin red staining, Carl Roth, Karlsruhe, Germany), respectively. (**B**) RNA expression levels for the adipogenic marker *FABP4* and the osteogenic marker *OPN* after 21 days in adipogenic differentiation medium (FABP4) or osteogenic differentiation medium (OPN) relative to untreated controls are shown. Total RNA was extracted from mesenchymal stem cells (MSC) as well as the SRH cell line. Quantitative RT-PCR was performed in quadruplicate and normalized to the house keeping gene *TBP*. Error bars indicate the standard deviation.

The SRH cell line was tested for its multilineage potential by inducing adipogenic and osteogenic differentiation. The adipogenic differentiated SRH cultures showed an increased accumulation of lipid droplets in the cells, as shown by Oil Red O and HCS LipidTOX™ Green staining (Figure 9A).

Osteogenic differentiation could be demonstrated by Alizarin red staining of a calcified bone matrix.

On day 21, quantitative RT-PCR of induced cultures showed clear signs of enhanced adipogenic and osteogenic marker expression (Figure 9B). *FABP4* expression was clearly elevated when compared to undifferentiated cultures, although the induction in MSC was twice as high as in SRH cells. The increase of *OPN* expression was comparable in MSC and SRH cells.

### 3.9. Transplantation into Mice

**Figure 10:**
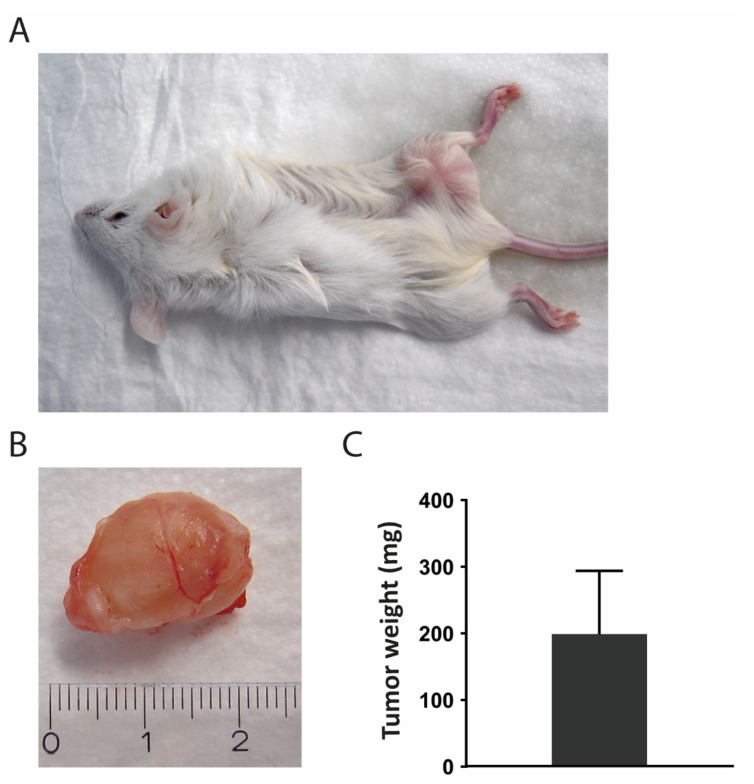
Mouse xenografts after subcutaneous injection of 5 × 10^6^ SRH cells. (**A**) Representative image showing a NSG mouse with a subcutaneous tumor nine weeks after inoculation. (**B**) Representative image of a removed tumor. (**C**) Dissected tumors were weighed and the mean weight (mg) with standard deviation was plotted.

For tumorigenicity assessment, the SRH cells were subcutaneously transplanted into three female NOD/SCID/γ_c_^null^ (NSG) mice (Figure 10A) and a tumor with a weight of 199 ± 95 mg was observed in all mice after 8–10 weeks (Figure 10B,C).

### 3.10. Cell Line Authentication

The authentication of the SRH cell line was performed by examination of STR loci of autosomal markers compared to the matching original tumor and the tumor xenograft (Table 2).

In addition, the presence of the AMEL locus confirmed the female identity of the patient tissue and cell line. The STR patterns of the SRH cell line were unique compared to any other cell line using the CLIMA v2.1 database. The xenograft was exclusively positive for the murine marker that was included in the StemElite™ ID System. 

## 4. Discussion

Cell lines are valuable model systems for the investigation of RMS genesis as well as preclinical examination of new treatment options. While several ERMS and ARMS cell lines are available for research [39], the establishment of the first SSRMS cell line from an adolescent was only recently reported [29]. Indeed, SSRMS is a rare RMS subtype that is often characterized by aggressive progress, especially when exhibiting a *MYOD1* mutation [16,17,18,19]. 

The primary tumor from which the SRH cell line was established showed the typical SSRMS morphology with spindle cells in fascicular patterns and scattered larger tumor cells in a sclerosing matrix [14,15,16,24]. Prevalent desmin positivity was found, irrespective of cell morphology, while nuclear MYOD1 reactivity was abundant, yet not ubiquitously present, features that have been already reported for other SSRMS tumors [14,15,16,24]. A TEM micrograph of a SRH cell depicted an elongated, irregular shaped nucleus. Glycogen storage characteristic for myogenic cells, but also a variety of cancer cell lines was present in SRH cells [40]. In addition, structures that resemble intermediate filaments, which are typical for muscle sarcomeres, could be detected [41]. 

Growth curves of SRH cells revealed a continuous growth with a doubling time of approximately 34 h, which is somewhat faster when compared to the SSRMS cell line established by Yoshimatsu et al. [29]. Most SRH cells (70%) were situated in the G1 phase of cell cycle. 

The karyotype of SRH cells revealed several translocations, deletions, and derivative chromosomes, especially affecting chromosomes 3, 6, 9, 10, 12, 18, and 21. Due to translocation of the long arm of chromosome 12 to chromosome 10 and 18, a tetraploidy of this part occurred. Complex chromosomal aberrations are typical for SSRMS, which are lacking a fusion gene [20,21,22]. Additionally, in 25–50% of ERMS chromosomal aberrations including gains of chromosome 2, 8, 12, and 13 can be observed [1,42]. In fusion gene, positive ARMS changes are typically restricted to amplifications of specific genomic regions without gross chromosomal alterations [42]. 

Array-CGH showed several gains and losses of individual chromosomal regions in SRH cells. With the exception of chromosome 12, which exhibited a large proportion of gains in q13–q15 and q21–q24, losses were dominating. To obtain an overview about the possible interactions of gene products located in chromosomal regions with aberrations an ingenuity pathway analysis (IPA) has been performed. Regarding pathways that are involved in stemness and differentiation, especially the WNT pathway of SRH cells was affected by loss of chromosomal regions coding for several components, while gains were basically restricted to regions coding for inhibitory proteins. In the NOTCH pathway, the *NOTCH1* and *HES7* genes were specifically located in chromosomal regions with losses. In contrast, in the Hh pathway, the transcription factor *GLI1* and *STK36* genes were located in regions with gains. Yet, *GLI1* mRNA and protein expression in SRH cells was rather restricted when compared with several ERMS and ARMS cell lines, some of which also bear amplifications of the corresponding genomic region 12q13.3 [31,43]. Similarly, ERMS and ARMS tumors frequently show amplifications of the corresponding region, including the adjacent *MDM2* gene, which is also covered by the gains in SRH cells [44]. Although NOTCH signaling has been implicated in RMS motility and stemness [27], this pathway is not significantly affected by chromosomal gains or losses in SRH cells. The downregulation of the WNT pathway concurrently with the activation of the Hh pathway has been likewise reported for ERMS and ARMS [27,28]. The impairment of WNT signaling concomitantly with *GLI1* overexpression probably contributes to the restraint of myogenic differentiation, which is also restricted by the *MYOD1* hotspot mutation that is present in SRH [16,17,18,19,27]. Remarkably, mRNA expression of the myogenic marker *MYMK*, *MYF5*, *MYOD1*, and *TANC1* was enhanced in SRH cells, whereas the expression of *MYOG* and especially *MYF6* mRNA was significantly decreased in SRH cells. The impaired myogenic differentiation despite the expression of myogenic transcription factors being a hallmark of all RMS subtypes [45] and it may by caused by different incidents including the presence of fusion proteins in ARMS or inhibiting *MYOD1* mutations. The substantial loss of *MYF6* mRNA expression is even more astonishing, since the chromosomal region coding for both *MYF5* and *MYF6* is amplified in SRH cells. However, *MYF6* is transactivated by several other myogenic transcription factors during late myogenesis with the highest impact of *MYOD1*, which may be one reason for the impairment of its expression [46,47]. The gene of Cyclin dependent kinase 4 (*CDK4*) implicated in G1 phase of cell cycle was located in an amplified genomic region, a feature of SRH cells that is shared by ARMS and ERMS [44,48]. Together with a loss of chromosomal regions coding for the genes of the CDK4 inhibitors *CDKN2B* and *CDKN2A* observed in SRH cells, G1 progression may be accelerated [49]. Yet, additionally, the gene of checkpoint kinase 2 (*CHEK2*), involved in a G1 cell cycle arrest [50], was located in a chromosomal region with gains. Moreover, the amplification of the chromosomal region coding for cyclin dependent kinase 2 associated protein 1 (*CDK2AP1*), a negative regulator of CDK2 [51], was revealed by array-CGH. Finally, the *CDK5* and ABL1 enzyme substrate (*CABLES*) 1 and 2 genes, which both negatively regulate CDK2 [52], were located in chromosomal regions with amplifications, which may decelerate entry into S phase. Because the gain or loss of a chromosomal region does not necessarily indicate the amplification of a specific gene located therein or a change of protein expression, the impact of these chromosomal alterations on SRH cell cycle and other pathways remains speculative. 

The two *TP53* missense mutations found in SRH cells interfere with p53 function [53]. The R273H and P309S mutations change p53 transactivation, yet, they do not prevent DNA binding [54]. Instead, these mutations apparently elicit a shift in p53 isoform expression [55]. In sporadic RMS, *TP53* mutations are found with a low frequency in about 5–15% of tumors [56,57,58]. For SSRMS, no *TP53* mutations have been reported previously [23]. While *TP53* mutations often increase p53 protein stability, the observed gain of the chromosomal region harboring the *MDM2* gene in SRH cells may lead to increased MDM2 protein abundance and the subsequent repression p53 protein amount [59]. Whether the observed p53 mutations of the SRH cell line were induced during multimodal chemotherapy or have occurred in the primary tumor prior to therapy is unknown. 

The SRH cell line is derived from a mesenchymal tumor that is reflected by cell surface expression of mesenchymal markers, including CD10, CD44, CD73, CD90, CD105, CD146, and CD166 concomitant with the lack of the hematopoietic lineage surface markers CD14, CD19, and CD45 [60,61,62,63]. CD10 expression is probably restricted to a subset of human MSC [62]. Interestingly, CD10 expression has been also confirmed on human muscle progenitor cells [64]. Evidence regarding CD34 expression in MSC is contradictory [65]. Indeed, CD34 positivity may be detected in different progenitor cells [66]. In contrast to two pediatric cases of spindle cell RMS with obvious CD34 expression [67], a low percentage of SRH cells showed a weak positivity for CD34, which may be attributed to a subpopulation with enhanced myogenic potential [68]. SRH cells were able to undergo adipogenic and osteogenic differentiation, in accordance with the mesenchymal origin and lack of terminal myogenic differentiation. A fact that has been already revealed in a previous publication [69]. 

CSC are associated with the formation, therapeutic resistance, and aggressiveness of sarcomas and other tumors [70,71]. SRH cell spheres exhibited mRNA expression of typical CSC markers, including *ALDH1*, *PROM1*, and multidrug transporters [72,73]. In addition, mRNA expression of other stemness markers, like *BMI1*, *MSI1*, *MYC*, *NANOG*, *POU5F1*, and *SOX2* [73], was upregulated in SRH spheres when compared to monolayer culture. The existence of stem cell populations positive for *POU5F1*, *NANOG*, *MYC*, and *SOX2* has been already shown for ERMS and ARMS [42]. PROM1 protein expression has been associated with poor overall survival in ERMS [25], whereas NANOG protein expression has been related with the self-renewal capacity of ERMS cells [74].

Tumor formation in vivo is a key property of cancer cell lines [75] interacting with other cell types, extracellular matrix (ECM), and biomechanical cues in humanized mouse models. Upon subcutaneous injection in NOD/SCID/γ_c_^null^ mice, SRH cells efficiently formed tumors underlining their tumorigenicity. 

In previous publications, it could be shown that the SRH cell line underwent apoptosis induction by arsenic trioxide (ATO) and lithium chloride (LiCl), though the sensitivity of SRH cells was restricted compared to other ERMS and ARMS cell lines tested [31]. 

In summary, the SRH cell line is the first adult SSRMS cell line available. We report the establishment, genetic, and molecular characterization of the SRH cell line, which is a useful tool for preclinical research for novel therapeutic strategies addressing the rare SSRMS tumor entity.

## Figures and Tables

**Table 1:** Quantitative real time PCR (RT-PCR) primer sequences.

Primer	Sequence (5′–3′)
*TBP* for	TGCACAGGAGCCAAGAGTGAA
*TBP* rev	CACATCACAGCTCCCCACCA
*YWHAZ* for	ACCGTTACTTGGCTGAGGTTGC
*YWHAZ* rev	CCCAGTCTGATAGGATGTGTTGG
*ABCB1* for	GCTGTCAAGGAAGCCAATGCCT
*ABCB1* rev	TGCAATGGCGATCCTCTGCTTC
*ABCC1* for	CCGTGTACTCCAACGCTGACAT
*ABCC1* rev	ATGCTGTGCGTGACCAAGATCC
*ABCG2* for	GTTCTCAGCAGCTCTTCGGCTT
*ABCG2* rev	TCCTCCAGACACACCACGGATA
*ALDH1A1* for	CGGGAAAAGCAATCTGAAGAGGG
*ALDH1A1* rev	GATGCGGCTATACAACACTGGC
*BMI1* for	GGTACTTCATTGATGCCACAACC
*BMI1* rev	CTGGTCTTGTGAACTTGGACATC
*KLF4* for	CATCTCAAGGCACACCTGCGAA
*KLF4* rev	TCGGTCGCATTTTTGGCACTGG
*LIN28* for	CCAGTGGATGTCTTTGTGCACC
*LIN28* rev	GTGACACGGATGGATTCCAGAC
*MSI1* for	GCTCAGCCAAAGGAGGTGATGT
*MSI1* rev	GCGTAGGTTGTGGCTTGGAAAC
*MYC* for	CCTGGTGCTCCATGAGGAGAC
*MYC* rev	CAGACTCTGACCTTTTGCCAGG
*MYF5* for	CAGTCCTGTCTGGTCCAGAAAG
*MYF5* rev	GTCCACTATGTTGGATAAGCAATC
*MYF6* for	CCCTTCAGCTACAGACCCAAAC
*MYF6* rev	TCCTTAGCCGTTATCACGAGCC
*MYOD* for	CTCCAACTGCTCCGACGGCAT
*MYOD* rev	ACAGGCAGTCTAGGCTCGACAC
*MYOG* for	AGTGCCATCCAGTACATCGAGC
*MYOG* rev	AGGCGCTGTGAGAGCTGCATTC
*MYMK* for	ATGCGTCACGACATCCTGGAGT
*MYMK* rev	CAATGGTCAGGACGCCGAACAT
*NANOG* for	CTCCAACATCCTGAACCTCAGC
*NANOG* rev	CGTCACACCATTGCTATTCTTCG
*POU5F1* for	CCTGAAGCAGAAGAGGATCACC
*POU5F1* rev	AAAGCGGCAGATGGTCGTTTGG
*PROM1* for	CACTACCAAGGACAAGGCGTTC
*PROM1* rev	CAACGCCTCTTTGGTCTCCTTG
*SOX2* for	GCTACAGCATGATGCAGGACCA
*SOX2* rev	TCTGCGAGCTGGTCATGGAGTT
*TANC1* for	GTGTGTCTGCTGACCAAGAAGG
*TANC1* rev	GACCACTCACAAGTCAGCAGGT

**Table 2:** Cell line authentication based on short tandem repeat (STR) profile.

	Original Tumor	SRH Cell Line	SRH Xenograft
AMEL	XX	XX	XX
CSF1P0	11,12	11,12	11,12
D13S317	11,11	11,11	11,11
D16S539	11,12	11,12	11,12
D21S11	29,31.2	29,31.2	29,31.2
D5S818	12,12	12,12	12,12
D7S820	8,11	8,11	8,11
TH01	9.3,9.3	9.3,9.3	9.3,9.3
TPOX	8,9	8,9	8,9
vWA	14,17	14,17	14,17

## Supplementary Materials

The following are available online at https://www.mdpi.com/2073-4409/9/12/2668/s1. Figure S1: Array-CGH results; Figure S2: Hh signaling pathway; Figure S3: NOTCH signaling pathway, Table S1: Array-CGH tabular results by probe name.
