# Supplementary material for: Establishment and Characterization of a Sclerosing Spindle Cell Rhabdomyosarcoma Cell Line with a Complex Genomic Profile
Source: Cells. 2020 Dec 11;9(12):2668. doi: 10.3390/cells9122668 (PMC7763666; doi:10.3390/cells9122668)
Supplement: Supplementary file 1 [file cells-09-02668-s001.zip › Supplementary Figures with caption.docx]

Supplementary Figures


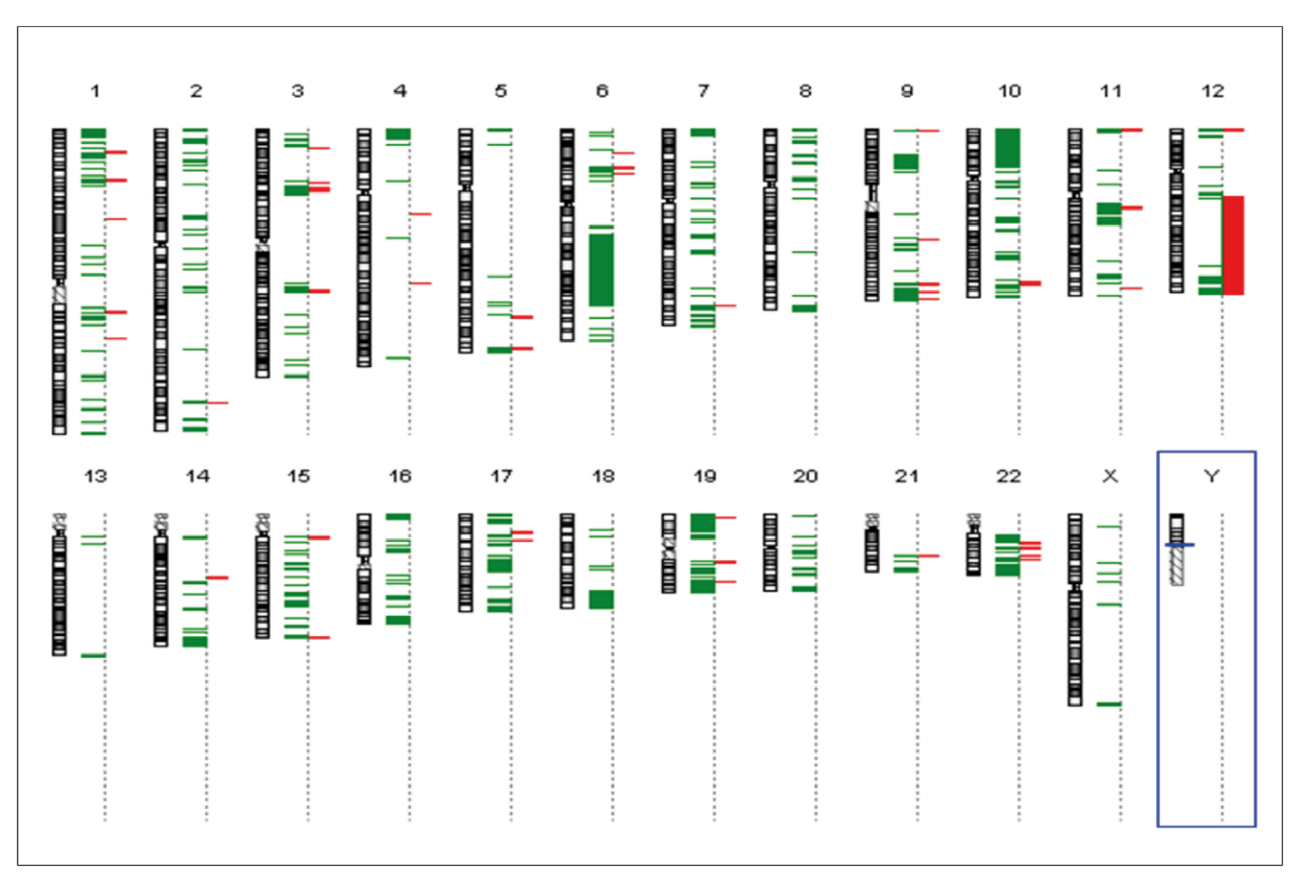


**Figure S1.** Array-CGH results. Whole genomic analysis of copy number changes in the SRH cell line compared to a reference DNA showed complex chromosomal rearrangements. Regions with copy number losses are indicated in green on left side, whereas regions with copy number gains are highlighted in red on the right side. Since the SRH cell line has been established from a female no Y chromosome is present (blue rectangle).


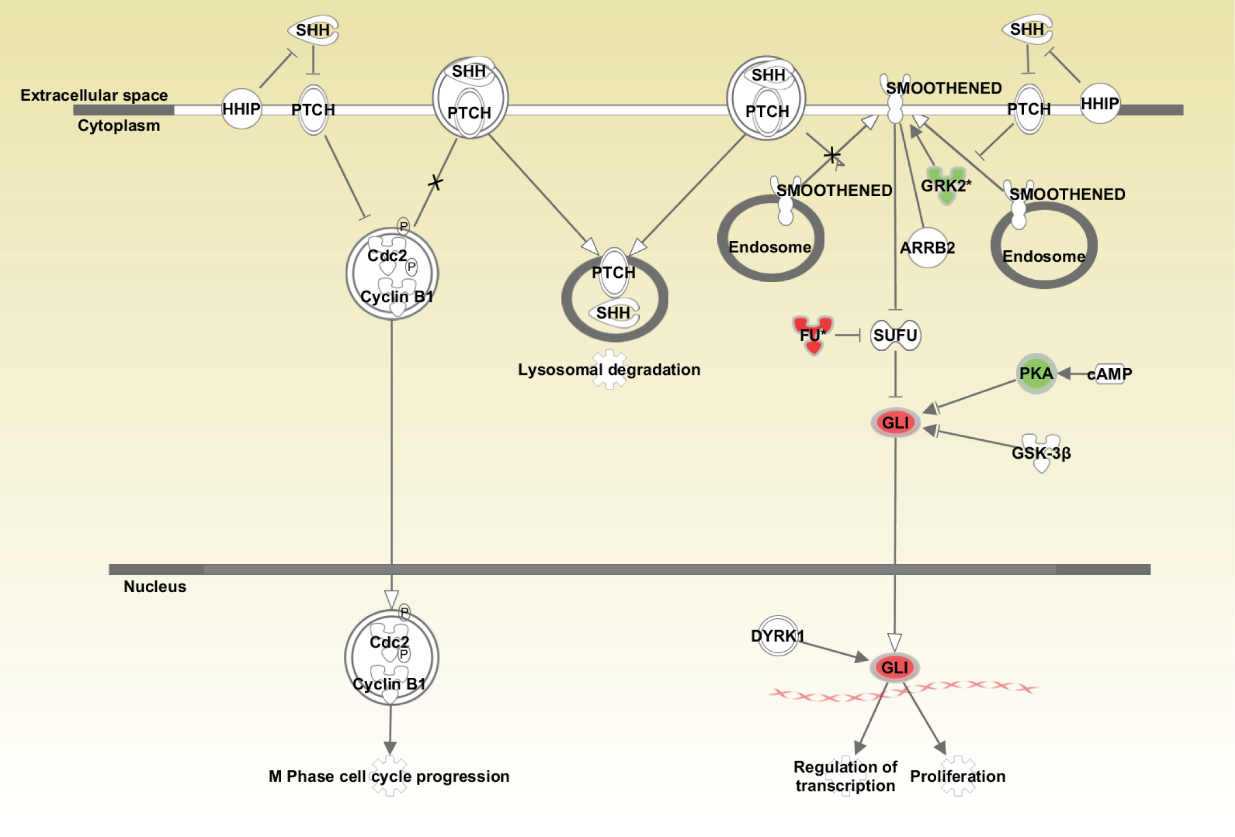


**Figure S2.** Hh signaling pathway. To investigate possible interactions of genes located in chromosomal regions with gains or losses, as determined by array-comparative genomic hybridization (CGH), ingenuity pathway analysis (IPA) has been performed. The chromosomal regions coding for genes that are shaded were determined to be significant from the statistical analysis. The genes shaded red are located in chromosomal regions with gains and those that are green are located in chromosomal regions with losses. White shaded genes relate to the pathway but were not found to be altered in the actual analysis. The intensity of the shading shows to what degree an amplification or loss of the specific chromosomal region was detected. A solid line represents a direct interaction between the two gene products, arrows show activating interactions, bar-headed lines inactivation.


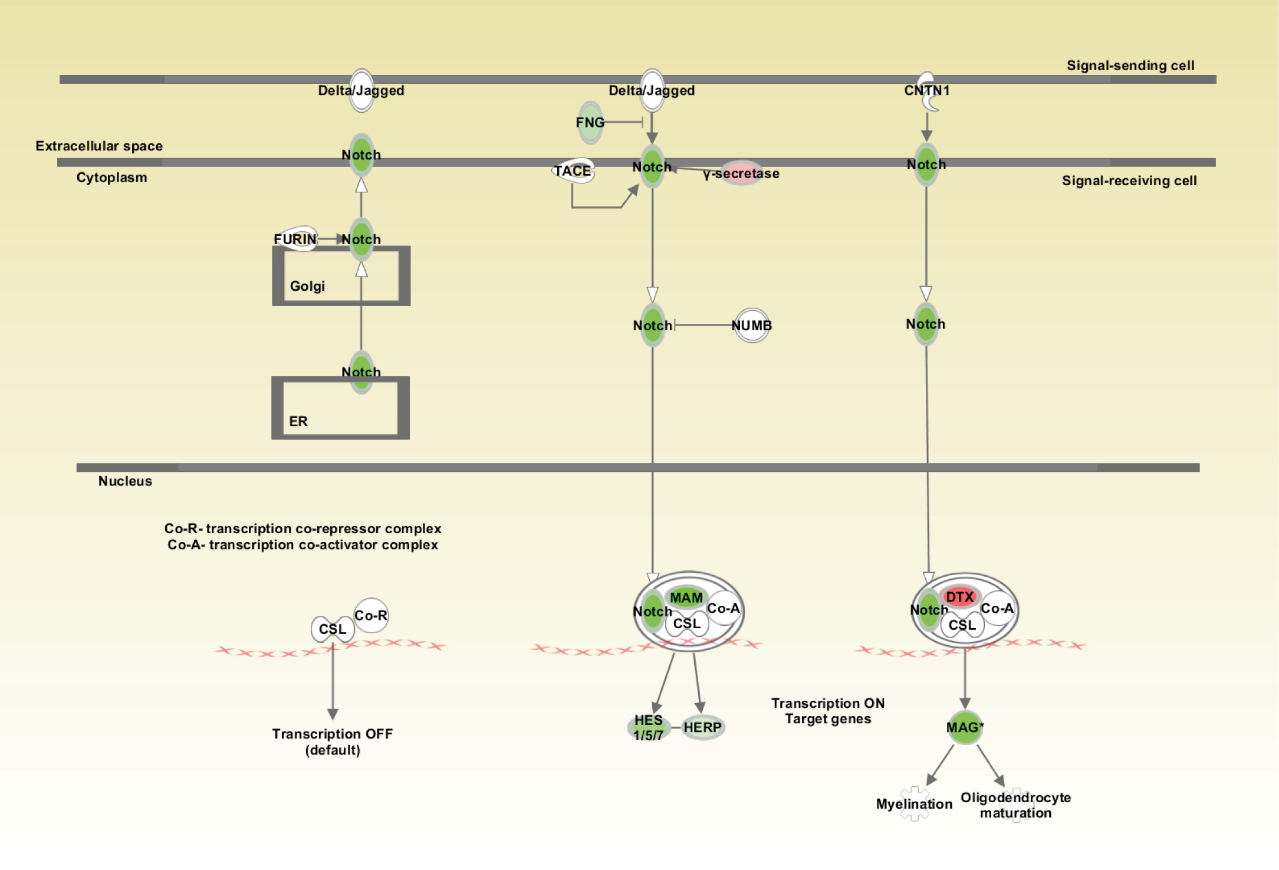


**Figure S3.** NOTCH signaling pathway. To investigate possible interactions of genes located in chromosomal regions with gains or losses, as determined by array-comparative genomic hybridization (CGH), ingenuity pathway analysis (IPA) has been performed. The chromosomal regions coding for genes that are shaded were determined to be significant from the statistical analysis. The genes shaded red are located in chromosomal regions with gains and those that are green are located in chromosomal regions with losses. White shaded genes relate to the pathway but were not found to be altered in the actual analysis. The intensity of the shading shows to what degree an amplification or loss of the specific chromosomal region was detected. A solid line represents a direct interaction between the two gene products, arrows show activating interactions, bar-headed lines inactivation.
